# The Quality of Life after Lymphaticovenous Anastomosis in 118 Lower Limb Lymphedema Patients

**DOI:** 10.1055/a-2117-4478

**Published:** 2023-09-08

**Authors:** Jin Geun Kwon, Yeongsong Kim, Min Young Jang, Hyunsuk Peter Suh, Changsik John Pak, Vaughan Keeley, Jae Yong Jeon, Joon Pio Hong

**Affiliations:** 1 Department of Plastic and Reconstructive Surgery, Asan Medical Center, University of Ulsan College of Medicine, Seoul, Republic of Korea; 2 Lymphoedema Service, Royal Derby Hospital, Derby, United Kingdom; 3 Department of Rehabilitation Medicine, Asan Medical Center, University of Ulsan College of Medicine, Seoul, Republic of Korea

**Keywords:** quality of life, lymphedema, patient-reported outcome measures

## Abstract

**Background** This is a prospective study on 118 patients who underwent lymphaticovenous anastomosis (LVA) due to secondary lower limb lymphedema between January 2018 and October 2020 to evaluate patients' quality of life (QOL) using the Quality of Life Measure for Limb Lymphedema (LYMQoL) questionnaire.

**Methods** The outcome measurement included the LYMQoL leg scoring system tool evaluating the function, appearance, symptom, mood, and overall outcome. In addition, correlation analysis was performed for three factors: based on International Society of Lymphology (ISL) stages, disease duration, and amount of volume reduction.

**Results** The LYMQoL tool overall satisfaction score significantly increased at all intervals from 4.4 ± 0.2 preoperative to 6.5 ± 0.3 postoperative at 12 months ( *p* < 0.001). Significant findings were seen for each domain scores compared preoperatively and at 12 months: function score (18.6 ± 0.5 to 15.4 ± 0.6), appearance score (17.8 ± 0.5 to 16.0 ± 0.6), symptom score (11.8 ± 0.3 to 8.9 ± 0.4), and mood score (14.5 ± 0.4 to 11.4 ± 0.5; *p* < 0.05). The correlation analysis between improvement of the overall score and the ISL stage ( *p* = 0.610, correlation coefficient [ *r* ] = − 0.047), disease duration ( *p* = 0.659, *r* = − 0.041), and amount of limb volume reduction ( *p* = 0.454, *r* = − 0.070) showed no statistical significance.

**Conclusion** The QOL of secondary lower limb lymphedema patients was significantly improved after LVA regardless of the severity of disease, duration of disease, and amount of volume reduction after LVA. Understanding the patient-reported outcome measurement will help the surgeons to manage and guide the expectations of the patients.

## Introduction

Lymphedema is a growing problem that may involve as much as 250 million patients worldwide that deteriorates the patient's quality of life (QOL). 1 Although etiology may vary among regions, most secondary lymphedema in the developed world is from cancer-related treatments. Lower limb lymphedema, in particular, often occurs after gynecological or genitourinary malignancies with an increased risk for patients undergoing pelvic dissections and radiation therapy. 2 3 4 The sense of heaviness, early tiredness, and other symptoms caused by swelling can limit the patient's daily activities. 5 In advanced cases, the skin begins to change causing fibrosis, leathery texture, elephantiasis (warty hyperkeratotic cobblestone appearance) which leads to the formation of fissures and discharge. Cellulitis may occur requiring admission and antibiotics use. 6 Mental health may be affected from dissatisfaction of appearance to reduced self-esteem. 7 8 Impaired sexual function from genital swelling or vaginal discharge may limit the patient's sexual life as well. 9 Most of all, lymphedema serve as a constant reminder of the cancer causing constant fear. 5 10 As mentioned, numerous studies have shown and continue to show that patients developing lymphedema exhibit high levels of not only functional but psychological, social, and sexual morbidity as well.

Therefore, in lymphedema patients, where cure is not possible, QOL measures are designed to enable patients' perspectives on the impact of health and health care interventions on their lives to be assessed and taken into account in clinical decision-making and research. 11 Furthermore, the change in patients' perception after surgical intervention will be an important factor to measure as most papers related to improvement focus on clinical improvement rather than the QOL. The reality often shows the discrepancy between patient satisfaction and improvement of symptoms such as reduction of limb circumference making it difficult to rationalize surgery from the patient's perspective. Thus, using patient-reported outcome measurement (PROM) studies to collect subjective information directly from the patient regarding specific or general conditions and add to clinical and functional outcomes and turn unmeasurable subjective qualities into quantitative measures will allow us to understand the impact of surgery. 12 13 The use of PROM in breast surgery, Breast-Q, has provided important insights highlighted by the literature concerning autologous reconstruction, implant type, fat grafting, and patient education. 14 15 Thus, to further understand the impact of lymphedema surgery, it will be prudent to have PROM studies.

Several PROMs have been developed for lymphedema patients, and among them, a Quality of Life Measure for Limb Lymphedema (LYMQoL) developed by Keeley et al is one of the widely used tool. 16 LYMQoL has the advantage of evaluating not only patient's overall satisfaction, but also measures satisfaction by subcategory of function, appearance, symptom, and mood. There have been few studies that have used LYMQoL and showed that surgical intervention such as lymphaticovenous anastomosis (LVA) has the potential to improve patients' QOL. 17 18 19 But the previous study have only a limited number of patients who participated in the study and lacks focus on the lower extremity. 17 18 19 The small number of participants also makes it difficult to obtain meaningful subcategory and subgroup analysis for further insight. 2 For example, will the response be different from patients who had long duration of lymphedema versus shorter duration or patients who had better volume reduction compared with the less?

The primary purpose of this study was to evaluate lower limb lymphedema patients' QOL using the LYMQoL questionnaire who underwent LVA. Furthermore, the relationship between patient's QOL survey outcome and lymphedema severity, duration, or amount of volume reduction after surgery was evaluated to gain further insight. This is the largest patient-enrolled study till date according to our knowledge.

## Methods

This is a prospective study on patients who underwent LVA for secondary lymphedema of the lower limb between January 2018 and October 2020. The study was approved by the institutional review board of Asan Medical Center. Exclusion criteria were (1) patients with combined liposuction or lymph node transfer, (2) follow-up of less than 1 year, (3) who submitted incomplete questionnaire, and (4) who refused to participate the study.

The demographic data included age, sex, marital status, urban–rural status, body mass index (BMI), comorbidity, etiology and extent of lymphedema, history of radiotherapy, time from cancer-ablation surgery/trauma to LVA, duration of lymphedema, history of cellulitis requiring admission, International Society of Lymphology (ISL) stage, and follow-up period. The surgical data included the mean number of performed LVA for the affected limb and the anastomosis method.

The Korean version of the LYMQoL lower leg tool was produced through a forward-backward translation process as recommended. 20 21 Seven translators participated in the forward translation procedure, and two translators participated in the backward translation procedure. The forward translation was performed by Korean-native speakers fluent in English. Translation was performed independently, and a consensus was reached. Backward translation was done by native English speakers fluent in Korean. After a consensus was reached on the two backward-translated versions, it was given to a Korean not working in the medical field to evaluate whether it contained any parts that were difficult to understand. At the end of this study, there was news that LYMQoL validation was being studied at another medical institution, and it was agreed that there was no significant difference between the version ongoing validation process and the version used in this paper.

The outcome measurement included LYMQoL leg scoring system tool surveyed at preoperative, postoperative 1 month, 6 months, and 12 months. LYMQoL tool is comprised of 27 questions for 4 domains (8 for function, 7 for appearance, 5 for symptom, 6 for mood domains, and 1 for overall score). 16 Regarding the score of function, appearance, symptom, and mood, lower score reflected better QOL. In contrast, the overall score on a scale of 10, higher score reflected better QOL. The postoperative improvement in the score for each domain and overall score were analyzed.

The volume of limb was calculated by using tape-measured circumference (5, 10, and 15 cm above and below the popliteal crease in standing position) applied to an equation (volume = π × H × (R ^2^ + r ^2^ + Rr)/3 (π = constant, H = height, R = radius [base], *r* = radius [top]). 22 23 The ratio of the reduction volume compared with the preoperative volume at each follow-up interval were calculated and used as an outcome parameter.

Correlation analysis was performed to see whether these three factors played a role in the outcome of LYMQoL tool: based on ISL stages, disease duration, and amount of volume reduction.

### Lymphaticovenous Anastomosis Protocol

The main indication for LVA is patients where lymphedema is resistant to volume reduction despite 2 months of intensive compressive therapy. 24 Regardless of the ISL stage, LVA was performed as a first-line surgical treatment when possible. 4 22 24 To identify functioning lymphatics and sizable superficial vein, magnetic resonance lymphangiography and high-frequency ultrasonography were used preoperatively. In addition, indocyanine green (ICG) and 0.2 cc of 10% fluorescein sodium (Fluorescite, Alcon, Fort worth, TX) dye were used intraoperatively for visualizing the functioning lymphatics. 25 The LVAs were performed on either side (lymphatic vessel)-to-end (vein) or end-to-end with a 11–0 nylon sutures. 26 After anastomosis, patency was confirmed based on intraluminal color change of the vein and confirmation from the flow of ICG.

### Statistical Analysis

A paired *t* -test was used to analyze the volume difference (postoperative 1 month and 6 months), while Wilcoxon test was used (postoperative 12 months) when there was a skewness at normality test. To analyze the longitudinal data of LYMQoL, linear mixed effects modeling (covariance pattern model) was performed. The pattern of covariance between repeated observations was modeled using a covariance pattern model to account for the correlation between the observations within the subject. A selection of covariance patterns was made by the comparison between the models using likelihood ratio tests. For the correlation analysis, Pearson correlation was used for disease duration and amount of volume reduction variables and Spearman correlation was used for ISL stage variable. All statistical analyses were performed using IBM SPSS version 21.0 (IBM Corp., Armonk, NY). *p* -Values < 0.05 were considered to indicate statistical significance.

## Results

Total of 118 patients were enrolled in this study. The average age of the patients was 54.4 ± 12.3 years, 92.4% were female, 80.5% were married, 83.9% lived in urban area and the average BMI was 24.9 ± 3.9 kg/m ^2^ . Bilateral lymphedema patients were seen in 19.5%, median duration of lymphedema was 48 months (IQR = 70 months), and 50% of patients had history of cellulitis requiring hospitalization. The distribution of ISL stages were 4.2%, 38.1, 31.4, and 26.3%, respectively, for stage I, II early, II late, and III. The number of LVA performed per limb was 3.4 ± 1.3. The LVA methods were 28.4, 37.8, and 33.8%, respectively, for end-to-end only, side (lymphatic vessel)-to-end (vein) only, and combined. The patient demographics are shown in Table 1 .

**Table 1:** Patient demographics

	Patient demographics ( *n* = 118)
Age, years	54.4 ± 12.3
Male/Female, *n* (%)	9 (7.6)/109 (92.4)
Single/Married status, *n* (%)	23 (19.5)/95 (80.5)
Urban/Rural status, *n* (%)	99 (83.9)/19 (16.1)
BMI, kg/m ^2^	24.9 ± 3.9
DM, *n* (%)	9 (7.6)
HTN, *n* (%)	26 (22.0)
Etiology, *n* (%)	
Cervix cancer	67 (56.8)
Ovarian cancer	16 (13.6)
Endometrial cancer	12 (10.2)
Other malignancy	14 (11.9)
Trauma	9 (7.6)
Uni-/bilaterality, *n* (%)	95 (80.5)/23 (19.5)
Etiology to LVA, months	143 ± 108.4
LE disease duration, months	median: 48 (IQR: 70)
Radiotherapy, *n* (%)	41 (34.7)
Cellulitis	59 (50.0)
ISL stage, *n* (%)	
I	5 (4.2)
II early	45 (38.1)
II late	37 (31.4)
III	31 (26.3)
Follow-up duration, months	27.3 ± 8.7
N of performed LVA	3.4 ± 1.3
LVA methods	
End-to-end only	23 (19.5)
Side-to-end only	63 (53.4)
Combined	32 (27.1)

Abbreviations: BMI, body mass index; DM, diabetes mellitus; Etiology to LVA, time period from cancer-ablation surgery or trauma to LVA; HTN, hypertension; ISL, International Society of Lymphology; LE, lymphedema; LVA, lymphaticovenous anastomosis; N, number.

**Fig. 1:**
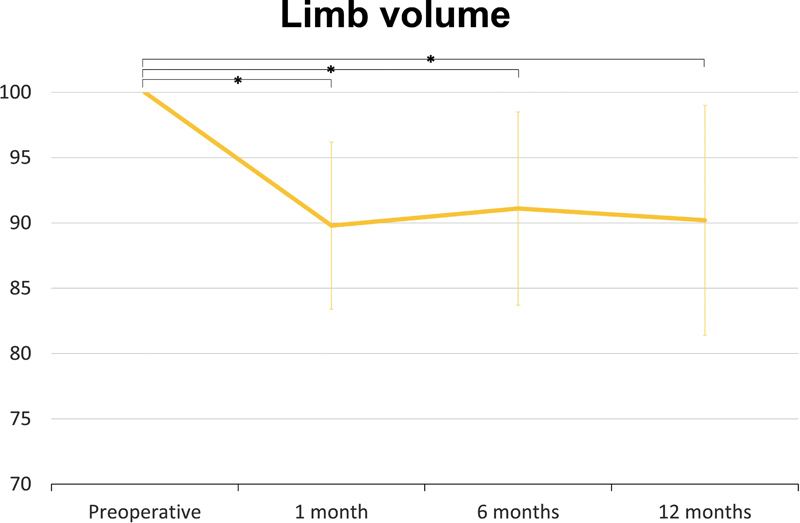
Ratio of volume reduction compared with the preoperative volume. There were 11.0 ± 6.6%, 10.4 ± 7.5%, and 11.7 ± 9.5% volume reduction, respectively, at postoperative 1 month, 6 months, and 12 months ( *p* < 0.001).

The ratio of volume reduced compared with the preoperative volume was 10.2 ± 6.4, 8.9 ± 7.4, and 9.8 ± 8.8%, respectively, at postoperative 1, 6, and 12 months ( *p* < 0.001; Fig. 1 ). The median value of volume reduction was 8%.

**Fig. 2:**
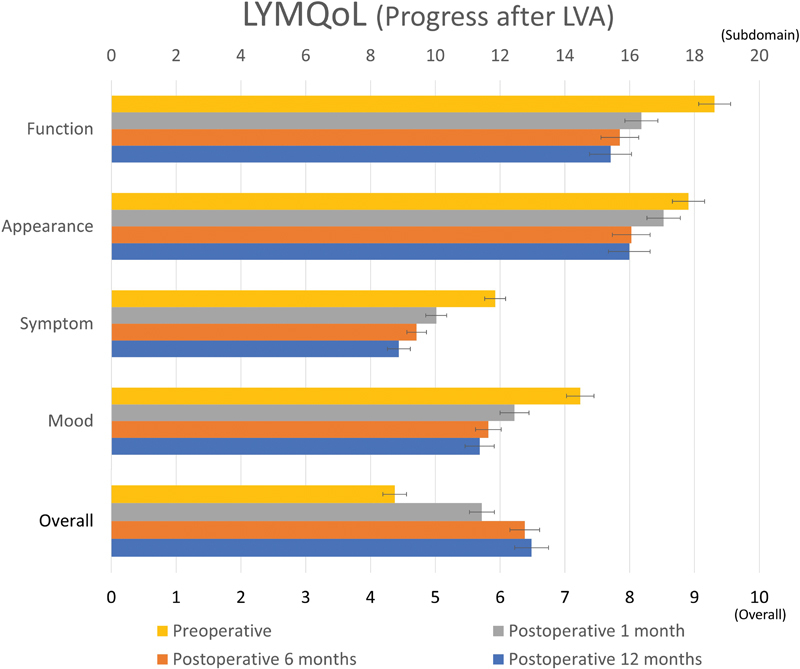
Lymphedema Quality of Life (LYMQoL) score after lymphaticovenous anastomosis (LVA). There was statistically significant improvement at function, appearance, symptom, and mood scores. Overall score was significantly increased from 4.3 ± 0.2 to 6.4 ± 0.3 at postoperative 12 months ( *p* < 0.001).

The LYMQoL tool overall satisfaction score significantly increased at all intervals measured 4.4 ± 0.2 at the preoperative survey reaching 6.5 ± 0.3 at postoperative 12 months ( *p* < 0.001) showing improved overall satisfaction. There was a statistically significant decrease in domain scores at LYMQoL questionnaire at 12 months; function score decreased from 18.6 ± 0.5 to 15.4 ± 0.6 ( *p* < 0.001), appearance score decreased from 17.8 ± 0.5 to 16.0 ± 0.6 ( *p* = 0.015), symptom score decreased from 11.8 ± 0.3 to 8.9 ± 0.4 ( *p* < 0.001), and mood score decreased from 14.5 ± 0.4 to 11.4 ± 0.5 ( *p* < 0.001). These decreased scores represent an improvement of each category ( Table 2 , Fig. 2 ). When evaluating each interval for function, symptom, and mood scores, they significantly decreased at postoperative 1, 6, and 12 months, while the appearance score significantly decreased only at postoperative 6 and 12 months.

**Table 2:** Progress pattern of Lymphedema Quality of Life (LYMQoL) score after lymphaticovenous shunt

	Preoperative	1 mo	6 mo	12 mo	*p* -Value(overall)
Function (range)(paired *p* -value) a	18.6 ± 0.5 (8–32)	16.4 ± 0.5 (8–32) (<0.0001)	15.7 ± 0.6 (8–29) (<0.0001)	15.4 ± 0.6 (8–32) (<0.0001)	<0.0001
Appearance(range)(paired *p* -value) a	17.8 ± 0.5 (7–28)	17.0 ± 0.5 (7–28) (0.0653)	16.0 ± 0.6 (7–27) (0.0005)	16.0 ± 0.6 (7–28) (0.0015)	0.0006
Symptoms(range)(paired *p* -value) a	11.8 ± 0.3 (5–20)	10.0 ± 0.3 (5–20) (<0.0001)	9.4 ± 0.3 (5–18) (<0.0001)	8.9 ± 0.4 (5–20) (<0.0001)	<0.0001
Mood(range)(paired *p* -value) a	14.5 ± 0.4 (6–24)	12.4 ± 0.4 (6–24) (<0.0001)	11.6 ± 0.4 (6–22) (<0.0001)	11.4 ± 0.5 (6–24) (<0.0001)	<0.0001
Overall(range)(paired *p* -value) a	4.4 ± 0.2 (0–9)	5.7 ± 0.2 (1–10) (<0.0001)	6.4 ± 0.2(1–10)(<0.0001)	6.5 ± 0.3 (1–10) (<0.0001)	<0.0001

a *p* -Value of comparison between preoperative volume and postoperative volume.

**Fig. 3:**
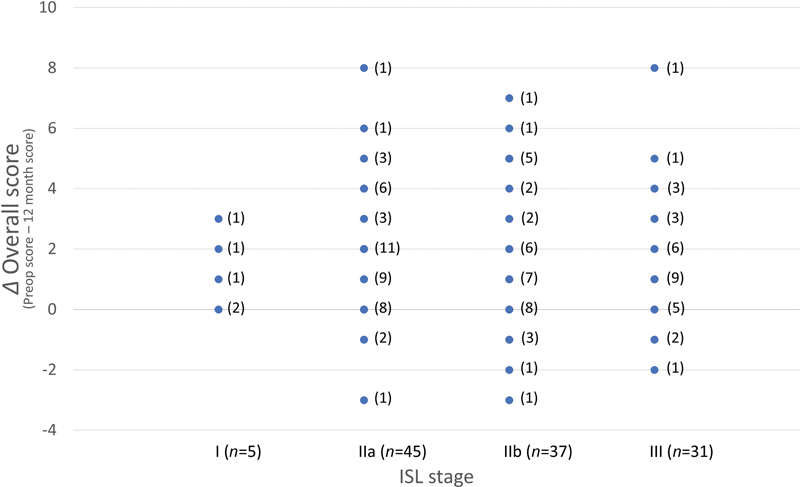
The relation between disease severity (international society of lymphology [ISL] stage) and the improvement of overall score at postoperative 12 months. There was no significant correlation between ISL stage and improvement of overall score ( *p* -value = 0.610). In this graph, the number of the patients corresponding to each plot is indicated in parentheses.

**Fig. 4:**
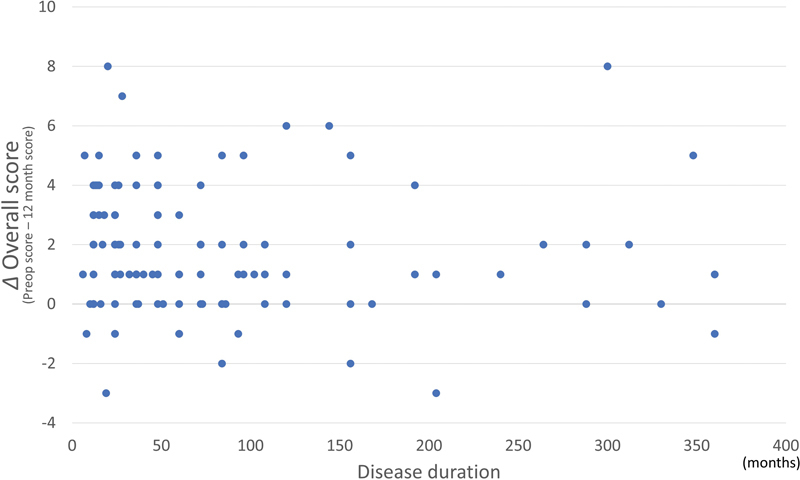
The relation between disease duration and the improvement of overall score at postoperative 12 months. There was no significant correlation between disease duration and improvement of overall score ( *p* -value = 0.659).

**Fig. 5:**
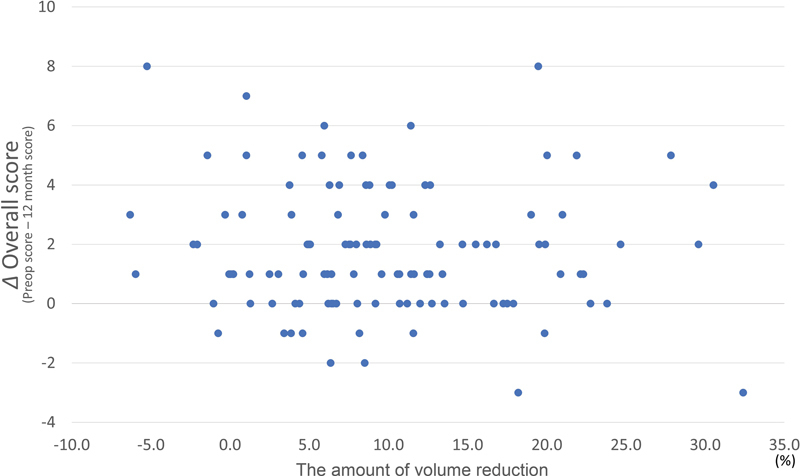
The relation between the amount of limb volume reduction and the improvement of overall score at postoperative 12 months. There was no significant correlation between the amount of limb volume reduction and improvement of overall score ( *p* -value = 0.454).

The outcome of the correlation analysis between the improvement of overall score and the ISL stage shows no statistical significance ( *p* = 0.610, correlation coefficient(r) = -0.047) ( Fig. 3 , Table 3 ; Supplementary Table S1 , available in the online version). In addition, there was no correlation between the improvement of overall score and disease duration ( *p* = 0.659, *r* = − 0.041) ( Fig. 4 , Table 3 ). There was no correlation between the improvement of overall score and the amount of limb volume reduction ( *p* = 0.454, *r* = − 0.070; Fig. 5 and Table 3 ).

**Table 3:** The outcome of correlation analysis between improvement of overall score at 12 months and the international society of lymphology (ISL) stage, disease duration, the amount of limb volume reduction

	*p* -Value	Correlation coefficient
ISL stage	0.610	−0.047
Disease duration	0.659	−0.041
Amount of limb volume reduction	0.454	−0.070

Note: Spearman correlation analysis was performed for analyzing ISL stage variable and Pearson correlation analysis was performed for analyzing disease duration and amount of limb volume reduction variables.

## Discussions

This is the first large study to evaluate QOL after LVA in patients with lower limb lymphedema. Currently, there is no wide consensus on a universal PROM tool and various ad hoc tools along with more validated tools have reported good outcomes for lymphedema. 7 27 However, even with using these, most of the reports focused on the upper limb lymphedema and lacks studies focused on the lower extremity. 2 19 27 Thus, this PROM study was designed to include a large patient pool who underwent LVA to evaluate the QOL and to further provide meaningful insight through correlation analysis and subgroup analysis.

Including bilateral lymphedema was a potential issue to be included in the study. However, the comparison between the unilateral group and the bilateral group was performed prior to the study. Both statistical analysis and pattern analysis show no difference between the unilateral group and the bilateral group. Therefore, both unilateral group and bilateral group were included in this study ( Supplementary Table S2 and Supplementary Fig. S1 , available in the online version).

In this study, the overall satisfaction score from the LYMQoL leg scoring system significantly improved after LVA (4.4 ± 0.2 at preoperative survey reaching 6.5 ± 0.3 at postoperative 12 months). The overall score increased as much as 2.1 ± 0.2 during 1 year, and it was similar to the 2.6 value from the study by Salgarello et al conducted with 26 patients. 17 Thus, the patients who underwent LVA have a significant improvement in overall QOL supporting this surgical approach. However, further evaluation showed a peculiar pattern for patients with stage I lymphedema. The overall satisfaction score was aggravated at postoperative 1 month despite the improvement of volume. In contrast, the overall score of ISL stage IIa, IIb, and III patients continued to improve throughout the different intervals ( Supplementary Fig. S2 , available in the online version). Although the overall score for stage I patients also improved after 6 months, it is noteworthy that the expectations of the patients with milder lymphedema may be different from the more advanced stage patients.

When looking into the specific domains for LYMQoL, each domain showed improvement over time but there was a difference in the time point for improvement. Significant improvement in the function, symptom, and mood domains was noted early from postoperative 1 month. However, the appearance domain did not show significant improvement till postoperative 6 months. Even though the most reduction occurred in the first month (10.2 ± 6.4% volume reduction), the patient's perception of improvement may take much longer. This may be because a high discrepancy between the expectations of the patient and surgeon may exist. Even on the basic understanding of what constitutes QOL, doctor, and patient perceptions may significantly diverge. 28 Therefore, based on these results, it will be necessary to explain in detail how the recovery progresses including patient perception of appearance. If QOL issues and the potentially divergent perspectives are not acknowledged and integrated into the patient's assessment, it can result in a lack of understanding about the efficacy of treatment or even lack of compliance.

In this study, three types of correlation analysis were performed according to the disease severity, disease duration, and the amount of limb volume reduction after LVA. There was no significant correlation between the improvement of overall LYMQoL score and disease severity or duration. The authors recently have reported on how to increase the success of LVA for advanced lymphedema patients by using enhanced preoperative imaging resulting in significant volume reduction. 22 25 Thus, successful LVA can meaningfully help in improving QOL regardless of the stage and duration of the disease. This similar finding was also reported in a systemic review looking at QOL after lymphedema surgery for the lower limb. 2 Finally, when looking at the correlation based on the amount of limb volume reduction, there was no significant correlation between the improvement of the overall score and the amount of volume reduction. Although one can assume that satisfaction may be closely related to the amount of improvement, there was no significant correlation between improvement of limb volume after LVA and QOL in this study. This is a peculiar finding after LVA. However, studies have shown conflicting reports in regard to the correlation between QOL and volume reduction. 29 30 31 32 Further evaluation will be needed to ultimately determine how volume reduction will play a role in improving the overall QOL. This study suggests that significant improvements are made in the QOL of patients after LVA based on multiple factors not only limited to but including the amount of lower limb volume reduction.

There are few limitations of this study. First, although this is the largest lower extremity lymphedema LYMQoL series to date, it will be ideal to have more patients enrolled to further evaluate and perform subgroup analysis. Second, the follow-up period of this study was limited to 1 year. This is based on our experience and studies that show most volume reduction occurs in the first few months. However, longer investigation up to several years will bring better insight into how the patient perception changes over long period of time. Third, there are too many variables that need to be taken into account such as BMI, smoking, compliance, etiology, and others. It would be ideal if these variables could be controlled but considering the diversity of clinical manifestation of lymphedema patients and the difficulty of the PROM study, it is hard to control these variables unless increasing the collected data by multiple folds. Even though this is the largest study to date, the number of patients is still insufficient to conduct a multivariated study. Therefore, a follow-up study based on more patients is warranted. Nevertheless, the data and result at this stage still provide a meaningful conclusion that may help the surgeons understand the patients' perspective.

The QOL of secondary lower limb lymphedema patients were significantly improved after LVA regardless of the severity of disease, duration of disease, and amount of volume reduction after LVA. Understanding the PROM will help the surgeons to manage and guide the expectations of the patients.
