# Supplementary material for: The Quality of Life after Lymphaticovenous Anastomosis in 118 Lower Limb Lymphedema Patients
Source: Arch Plast Surg. 2023 Sep 8;50(5):514–22. doi: 10.1055/a-2117-4478 (PMC10556310; doi:10.1055/a-2117-4478)
Supplement: Supplementary file 1 — Supplementary Material [file 10-1055-a-2117-4478-s22oct0186oa.pdf]

Supplementary Table S1 Subgroup analysis based on the International Society Of Lymphology Stage (ISL stage) regarding the progress of Lymphedema Quality of Life (LYMQoL) score after lymphaticovenous shunt

	ISL stage	Preoperative	12 mo	Interaction[a]
Function	I	18.6 ± 2.4	18.0 ± 3.6	0.1161
	IIa	18.0 ± 0.9	14.4 ± 0.9	
	IIb	18.6 ± 0.9	14.8 ± 1.3	
	III	19.6 ± 1.0	16.1 ± 1.6	
Appearance	I	18.4 ± 2.4	17.6 ± 2.5	0.7065
	IIa	16.9 ± 0.8	14.6 ± 0.9	
	IIb	17.8 ± 0.9	16.3 ± 1.3	
	III	19.1 ± 1.0	18.0 ± 1.5	
Symptoms	I	14.6 ± 1.6	8.9 ± 0.9	0.3621
	IIa	11.6 ± 0.5	8.5 ± 0.5	
	IIb	12.1 ± 0.6	9.7 ± 0.7	
	III	11.6 ± 0.6	8.9 ± 1.4	
Mood	I	16.0 ± 2.1	10.9 ± 1.7	0.2115
	IIa	14.3 ± 0.7	11.3 ± 0.6	
	IIb	14.7 ± 0.8	11.2 ± 0.9	
	III	14.2 ± 0.8	12.4 ± 1.1	
Overall	I	5.2 ± 0.9	6.9 ± 1.0	0.4808
	IIa	4.2 ± 0.3	6.3 ± 0.4	
	IIb	4.5 ± 0.3	6.8 ± 0.5	
	III	4.3 ± 0.4	6.3 ± 0.7	

[a] < 0.05 means that there is a statistical significant difference between subgroups.

Supplementary Table S2 Comparison between the uniltaral group and bilateral group regarding the progress of Lymphedema Quality of Life (LYMQoL) Score after lymphaticovenous shunt

	Bilaterallity	Preoperative	1 mo	6 mo	12 mo	Interaction[a]
Function	Unilateral	18.5 ± 0.5	15.6 ± 1.0	14.1 ± 1.6	15.9 ± 0.6	0.9492
	Bilateral	19.4 ± 1.1	17.5 ± 1.8	16.1 ± 3.0	16.7 ± 1.3	
Appearance	Unilateral	17.6 ± 0.6	15.6 ± 1.1	12.9 ± 1.7	16.1 ± 0.6	0.5205
	Bilateral	18.6 ± 1.2	18.6 ± 1.9	17.7 ± 3.1	17.8 ± 1.3	
Symptoms	Unilateral	11.8 ± 0.4	10.0 ± 0.7	7.3 ± 1.2	9.5 ± 0.4	0.8549
	Bilateral	12.0 ± 0.7	10.7 ± 1.3	9.1 ± 2.2	10.4 ± 0.9	
Mood	Unilateral	14.4 ± 0.5	11.0 ± 0.9	9.6 ± 1.5	11.7 ± 0.5	0.7008
	Bilateral	14.7 ± 1.0	13.2 ± 1.6	11.7 ± 2.7	12.6 ± 1.1	
Overall	Unilateral	4.5 ± 0.2	5.7 ± 0.4	7.5 ± 0.7	6.2 ± 0.2	0.8111
	Bilateral	4.1 ± 0.4	5.2 ± 0.8	6.4 ± 1.3	6.3 ± 0.5	

[a] < 0.05 means that there is a statistical significant difference between subgroups.

 © 2023. The Author(s).

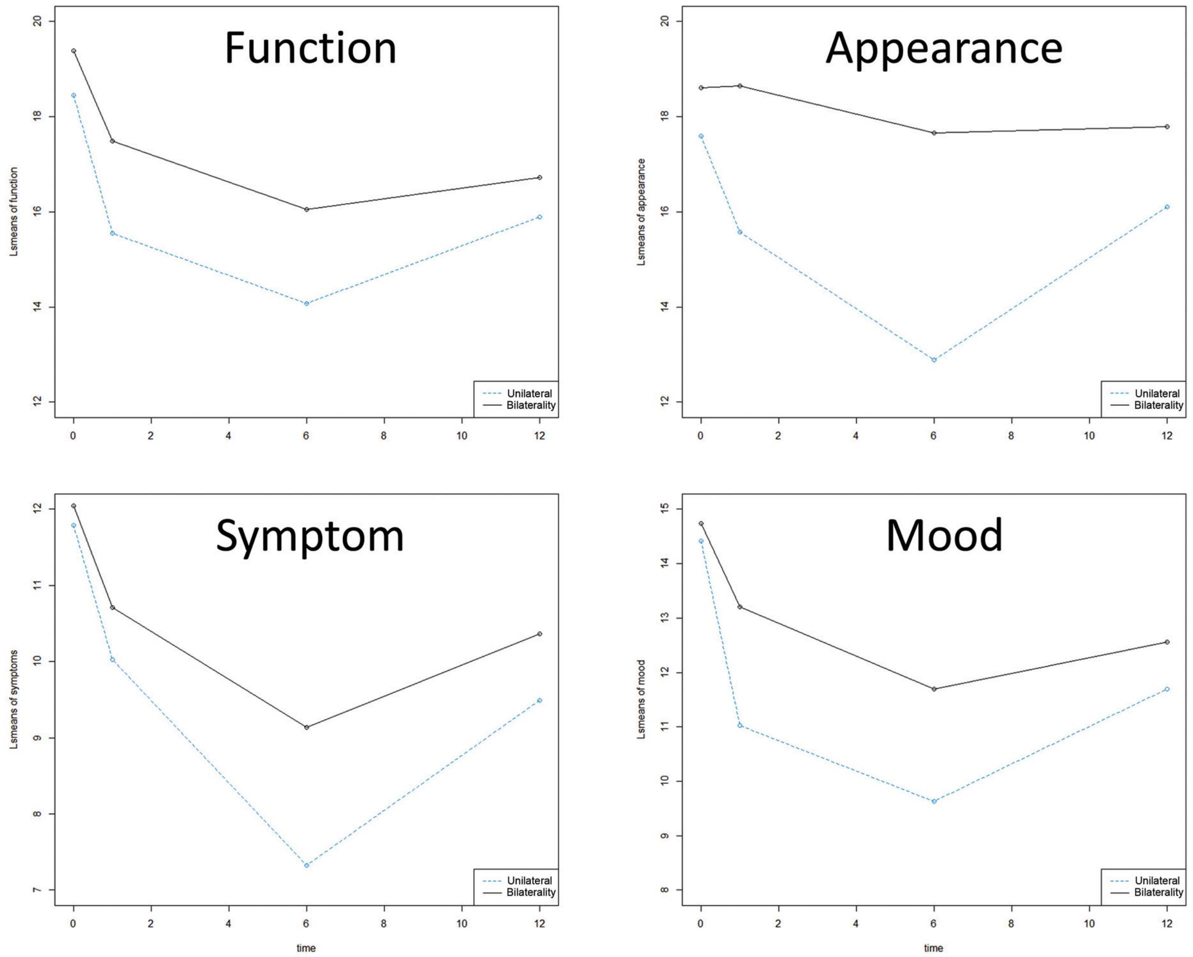

Supplementary Fig. S1 The progress pattern of unilateral group and bilateral group.

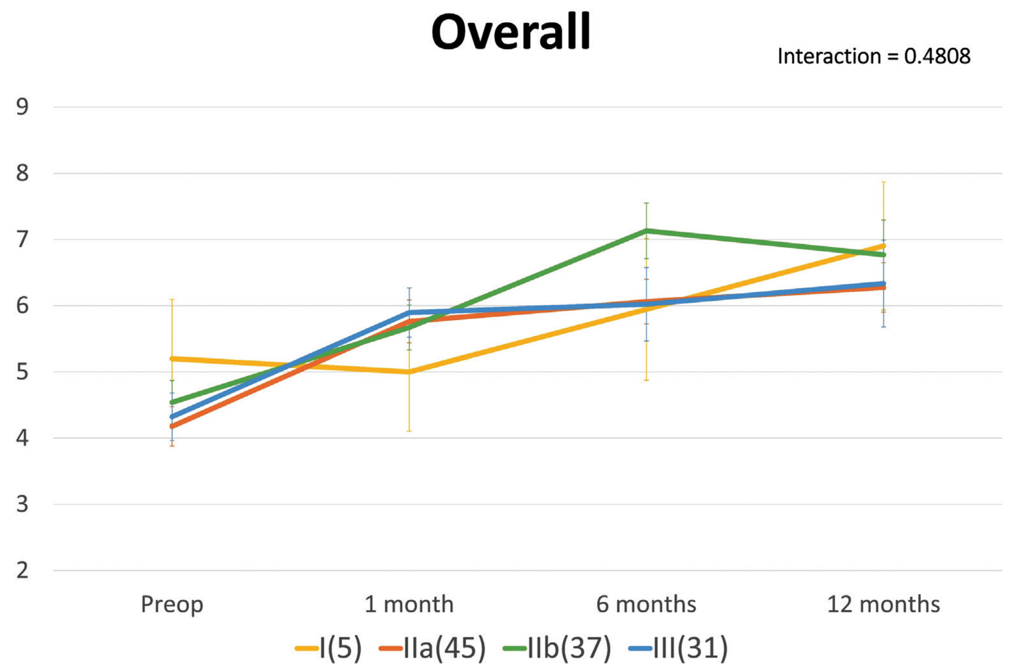

Supplementary Fig. S2 The progress pattern of overall score according to the International Society of Lymphology (ISL) stage.

 © 2023. The Author(s).
